# The effect of reverse transcription enzymes and conditions on high throughput amplicon sequencing of the 16S rRNA

**DOI:** 10.7717/peerj.7608

**Published:** 2019-10-25

**Authors:** Adam Šťovíček, Smadar Cohen-Chalamish, Osnat Gillor

**Affiliations:** 1 Department of Environmental Hydrology and Microbiology, Ben-Gurion University of the Negev, Beer Sheva, Israel; 2 The Mina and Everard Goodman Faculty of Life Sciences and Advanced Materials and Nanotechnology Institute, Bar-Ilan University, Ramat-Gan, Israel

**Keywords:** Reverse transcription, Methodology, Amplicon sequencing, RNA, TGIRT, ImProm-II, SuperScript, RT, Illumina, Ribosome

## Abstract

It is assumed that the sequencing of ribosomes better reflects the active microbial community than the sequencing of the ribosomal RNA encoding genes. Yet, many studies exploring microbial communities in various environments, ranging from the human gut to deep oceans, questioned the validity of this paradigm due to the discrepancies between the DNA and RNA based communities. Here, we focus on an often neglected key step in the analysis, the reverse transcription (RT) reaction. Previous studies showed that RT may introduce biases when expressed genes and ribosmal rRNA are quantified, yet its effect on microbial diversity and community composition was never tested. High throughput sequencing of ribosomal RNA is a valuable tool to understand microbial communities as it better describes the active population than DNA analysis. However, the necessary step of RT may introduce biases that have so far been poorly described. In this manuscript, we compare three RT enzymes, commonly used in soil microbiology, in two temperature modes to determine a potential source of bias due to non-standardized RT conditions. In our comparisons, we have observed up to six fold differences in bacterial class abundance. A temperature induced bias can be partially explained by G-C content of the affected bacterial groups, thus pointing toward a need for higher reaction temperatures. However, another source of bias was due to enzyme processivity differences. This bias is potentially hard to overcome and thus mitigating it might require the use of one enzyme for the sake of cross-study comparison.

## Introduction

Massively parallel amplicon sequencing revolutionized our view of microbial world: by sequencing a taxonomic tag such as 16S rRNA encoding gene, it allows taxonomic description of the microbial communities (Quammen, 2018). However, the existing approaches introduce caveats: the DNA amplicon sequencing may capture “relic DNA,” which is a recalcitrant genetic material from dead cells or naked DNA (Carini et al., 2016) in addition, amplicon sequencing carries technical biases due to sample preparation, DNA extraction methods (Pan et al., 2010), amplification reaction (Pfeiffer et al., 2014) and analysis (Pollock et al., 2018). Moreover, DNA-based microbiome information can describe the total community, but it cannot report which members are metabolically active (Blazewicz et al., 2013). In contrast to DNA-based tools, analysis of ribosomes can describe the metabolically active members of a given community. The combination of data generated from rRNA encoding genes and ribosomes led to a wide range of ecological insights, including the response to climatic changes (Angel et al., 2013), pH and water availability (Romanowicz et al., 2016), and biogeochemical processes (Freedman et al., 2015).

Ribosomal analysis studies are based on an assumption that ribosomes are more abundant in active cells compared to dormant ones (Blazewicz et al., 2013; Lennon & Jones, 2011). However, this assumption may not always be correct. Dormant bacteria may be misclassified as active, when ribosomes are present in cells and spores that are inactive (Segev et al., 2013; Blagodatskaya & Kuzyakov, 2013). In contrast, active bacteria with low metabolic turnover and low ribosomal count could be labeled as dormant when sequencing depth is insufficient (Steven et al., 2017; Joergensen & Wichern, 2018). In spite of various biases that introduce discrepancies in the community structure (Forney, Zhou & Brown, 2004), ribosomal analysis can capture the biological variability highlighting large differences between samples. However, if more subtle differences are of interest, technical biases could confound biological interpretations (Lever et al., 2015; McCarthy et al., 2015). This is due to specific challenges introduced RNA-based analysis (Bustin & Nolan, 2004, 2017). Therefore, to confidently compare results across ribosome-based amplicon sequencing studies, we must determine which component of the analysis: RNA extraction, processing or data analysis may influence the outcome and introduce biases.

Prior studies focused on biases in the steps of RNA extraction, amplification and sequencing, but disregard any biases that may occur during RT (Creer et al., 2016). At the crucial step of RT, most researches simply “follow the manufacture instructions” (Table 1). However, RT kits typically detail a wide range of temperatures, primer, template and reaction options, which may lead to different results. The reverse transcriptase enzyme requires sequence priming to initiate a reaction. Primers could be poly-A complementary, random or sequence specific. Poly-A priming is limited to eukaryotic mRNA which makes it unsuitable for use with ribosomal taxonomic tags. Opinions vary about the usefulness of random and sequence-specific priming for the analysis of microbiomes: Random priming may produce higher yield of cDNA and improve the detection limit (Zhang & Byrne, 1999; Ståhlberg et al., 2004), but may decrease the reproducibility and introduce bias (Bustin & Nolan, 2004; Hansen, Brenner & Dudoit, 2010). Sequence specific primes require fine tuning of the reaction conditions and higher template concentration than random priming (Ståhlberg, Kubista & Michael, 2004). Moreover, the results of the RT reaction is determined not only by the type of the RT enzyme used, but also by the reaction conditions (Curry, McHale & Smith, 2002; Ståhlberg et al., 2004; Ståhlberg, Kubista & Michael, 2004; Bustin & Nolan, 2004; Sieber et al., 2010).

**Table 1:** Literature overview of RT conditions applied in soil microbiological studies.

Manufacturer	RT enzyme	RT origin	Temperature (°C)		RNA type	Primer type	References
			Suggested	Used			
Promega	MMLV	MMLV	37–42	NA	rRNA	926R	Carson et al. (2010)
				NA	rRNA & mRNA	Random hexamers	Pratscher, Dumont & Conrad (2011)
	ImProm-II	AMV	37–55	42	rRNA & mRNA	Random hexamers	Angel et al. (2013)
				NA	rRNA	Random hexamers	Ke, Lu & Conrad (2015)
Qiagen	QuantiTect	Quantiscript	42–50	NA	rRNA	Unique RT Primer Mix	Barnard, Osborne & Firestone (2015)
				37	rRNA	Random hexamers	Placella, Brodie & Firestone (2012)
	Omniscript	Quantiscript	37	NA	mRNA	Random hexamers	Paulin et al. (2013)
				NA	mRNA	invA-R	García et al. (2010)
Takara	PrimeScript II	AMV	42–50	NA	mRNA	Random hexamers	Huang et al. (2016)
				NA	rRNA	Random hexamers	Che et al. (2018)
Roche	Roche reverse transcription kit	AMV	42–60	42 & 50	rRNA	Random hexamers	Nunes et al. (2018)
				42 & 50	rRNA	Random hexamers	Jurburg et al. (2017)
Thermo Fisher	MMLV	MMLV	37–42	45	rRNA	900R	Lillis, Doyle & Clipson (2009)
				NA	rRNA	Random hexamers	Baldrian et al. (2012)
	SuperScript-II	MMLV	42–55	NA	rRMA	1492R	Degelmann et al. (2009)
				NA	mRNA	Random hexamers	Nacke et al. (2014)
	SuperScript-III	MMLV	42–55	NA	rRNA	Random hexamers	Angel & Conrad (2013)
				NA	rRNA	27F & LR3	Romanowicz et al. (2016)

Ideally RT efficiency is near 100%, but in practice it varies dramatically: 90% efficiency was reported for SuperScript III (mutated MMLV RT) (Ståhlberg et al., 2004), 20% for murine leukemia virus (M-MLV) RT (Curry, McHale & Smith, 2002), and as low as 2% for Avian Myeloblastosis Virus (AMV) RT (Ståhlberg et al., 2004). Moreover almost two orders of magnitude difference were reported between mutated and wild-type AMV RT (Ståhlberg et al., 2004).

To the best of our knowledge no study has yet compared the RT reaction conditions for environmental microbiome profile. We hypothesize that during RT reactions, varying RT enzyme types and temperature conditions will yield different results in microbial diversity and community composition. We further predict that variations in communities will be G-C dependent. To test our prediction, we present a comparative study of commonly used RT enzymes in the field of environmental microbiology as well as a comparison of two different reaction temperatures.

## Materials and Methods

### Study site and sample collection scheme

Soil samples were collected at the central Negev Desert highlands, Israel (Zin Plateau, 30°86′N, 34°80′E) at an established ecological research site. The mean annual precipitation at the sampling site is 90 mm and the mean annual temperature is 30° (LTER data). Samples were collected under the canopy of perennial shrub *Hammada scoparia* in October 2015 at the end of the dry season as previously described (Baubin et al., 2019). Briefly, sampling was conducted in seven random blocks, each providing two technical replicates resulting in 14 samples. Samples were collected from the top five cm of the soil, following the removal of crust and debris. The soil samples were processed within 24 h of collection. Samples were homogenized using two mm sieve and the duplicates from each block were composited. This resulted in seven final replicates.

### RNA preparation

Total RNA was extracted from each of the seven samples using a phenol-chlorophorm extraction previously described by Angel (2012). The reaction buffer pH was adjusted to 5. The total RNA was subsequently purified with the MagListo™ Total RNA Extraction Kit (Bioneer, Daejeon, Republic of Korea). Contaminant DNA was removed using a DNAse I from the MasterPure RNA Purification Kit (Epicenter, Madison, WI, USA) with two successive treatments of 30 min according to manufacturer’s instructions. The reaction mixture was purified using the MagListo™ Total RNA Extraction Kit (Bioneer, Daejeon, Republic of Korea). The absence of contaminant DNA was verified using total bacterial primers 341F (5′-CCTACGGGAGGCAGCAG-3′) and 515R (5′-TTACCGCGGCTGCTGGCAC-3′) (Klindworth et al., 2013) and DreamTaq DNA polymerase (Thermo Scientific, Waltham, MA, USA) under the following conditions: 95 °C for 5 min, followed by 26 cycles of 95 °C for 15 s, 60 °C for 30 s and 72 °C for 30 s for extension followed by 72 °C for 5 min for final extension. If amplification was detected the sample was discarded, re-extracted, purified, and tested. Only DNA-free samples were used in this study.

### Reverse transcription reaction conditions

**Figure 1:**
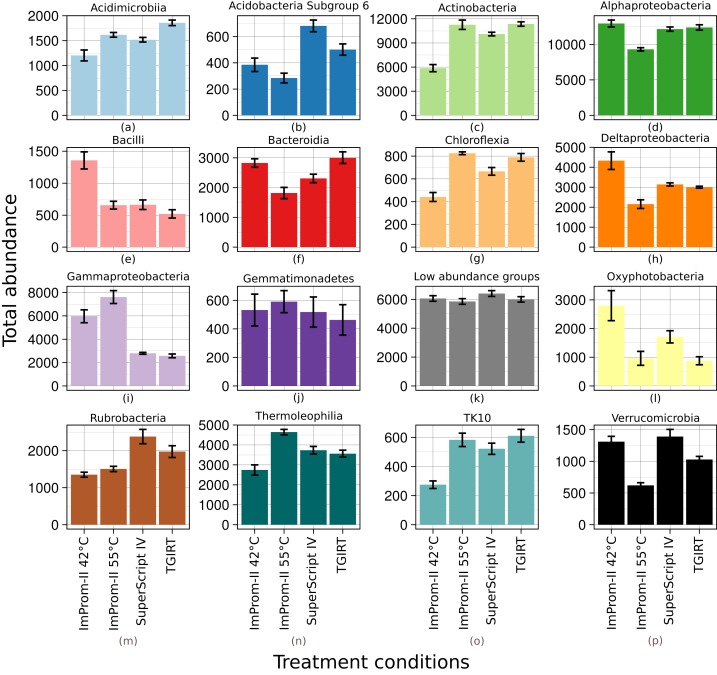
Relative abundance of main classes across each tested condition (A–P). Only top 15% of the most abundant classes are represented and the rest is summarized in the “Low abundance groups” category (K). The *x*-axis shows different enzymes and conditions. The *y*-axis shows an average relative abundance. Each category is an average of four samples.

RT kits used in this experiment were chosen to represent the most commonly used enzymes in the field (Fig. 1). All seven samples were reverse transcribed by the same kit to reduce batch effects. Each RT kit used in this study originated from a different source: (I) ImProm-II Reverse Transcription System enzyme (Promega, Madison, WI, USA) originates from AMV RT, (II) SuperScript IV Reverse Transcriptase Kit enzyme (ThermoFisher Scientific, Waltham, MA, USA) originates from MMLV RT and (III) TGIRT originates from the mobile group II introns reverse transcriptase (Mohr et al., 2013) TGIRT™-III Enzyme (InGex, St. Louis, MO, USA). Each reaction consisted of 50 ng of total RNA template, measured by Quanti-iT™ RNA Assay Kit (ThermoFisher, Waltham, MA, USA), and random hexamer primers (0.5 μg/reaction). Template and primer mix were heated to 70 °C (ImProm-II) or 65 °C (SuperScript IV). Each reaction was subsequently cooled to 4 °C for 5 min and incubated at 42 °C (ImProm-II), 55 °C (ImProm-II and Superscript) or 57 °C (TGIRT) for 60 min (ImProm-II), 120 min (TGIRT), or 10 min (SuperScript IV). All reactions were terminated and DNA was removed by alkaline lysis using 2 μL of 1M NaOH, incubating for 12 min at 70 °C. After which the reaction was neutralized using 4 μL of 0.5M acetic acid (Table 2).

**Table 2:** A summary of conditions applied to the different reaction conditions.

Manufacturer	RT kit	Primers	Thermo cycling		Reaction mix	
			Temperature (°C)	Time (min)	Reactant	Amount
Promega	Im-Prom II	Random Hexamers (500 ng/reaction)	70	5	DTT	10 μM
			4	5	Tris–HCl	50 mM
			25	5	KCl	75 mM
			42	60	MgCl_2_	2.5 mM
			70	15	dNTP	0.5 mM
					RNAse inhibitor	0.5/20 μL
Promega	Im-Prom II	Random Hexamers (500 ng/reaction)	70	5	DTT	10 μM
			4	5	Tris–HCl	50 mM
			25	5	KCl	75 mM
			55	60	MgCl_2_	2.5 mM
			70	15	dNTP	0.5 mM
					RNAse inhibitor	0.5/20 μL
Thermo Fisher	SuperScriptIV	Random Hexamers (2.5 μM)	65	5	DTT	5 μM
			0	1	Tris–HCl	50 mM
			23	10	KCl	50 mM
			55	10	MgCl_2_	4 mM
			80	10	dNTP	0.5 mM
					RNAse inhibitor	0.5/20 μL
TGIRT	TGIRT-III	Random Hexamers (500 ng/reaction)	65	5	DTT	5 μM
			0	1	Tris–HCl	10 mM
			23	10	EDTA	1 mM
			58	120	MgCl_2_	4 mM
			80	10	dNTP	0.5 mM
					RNAse inhibitor	0.5/20 μL

### Illumina sequence preparation

The V3 and V4 regions of the resulting cDNA were amplified using 341F (5′-CCTACGGGAGGCAGCAG-3′) and 806R (5′-GGTCTGGACTACHVGGGTWTCTAAT-3′) (Klindworth et al., 2013) primers. Each reaction was performed in triplicate and consisted of 1 mM bovine serum albumin (Takara, Kusatsu, Japan), 2.5 μL of 10× standard buffer, five μM primers, 0.8 mM dNTPs, 0.4 μL DreamTaq DNA polymerase (Thermo Scientific, Waltham, MA, USA), and 4 μL of template cDNA. The reaction mixtures were subsequently amplified using the following PCR conditions: 95 °C for 30 s, 27 cycles of 95 °C for 15 s, 50 °C for 30 s, 68 °C for 30 s, and 68 °C for 5 min. Resulting amplicon presence was verified using 1.5% agarose gel electrophoresis. Resulting technical triplicates were combined, and the sequencing libraries were constructed using the TruSeq^®^ DNA Sample Preparation Kit (Illumina, San Diego, CA, USA) following the manufacturer’s recommendations. The amplicon libraries were sequenced (250 × 2 base pairs, pair-end) on the Illumina MiSeq System platform at the Research Resources Centre at the University of Illinois.

### Sequence analysis

Resulting paired end sequences were merged using the CASPER program (Kwon, Lee & Yoon, 2014), and the resulting merged reads were clustered using the UPARSE pipeline according to the recommended settings (Edgar, 2013). The resulting operational taxonomic unit (OTU) representative sequences were taxonomically assigned with the SINA incremental aligner using a lowest common ancestor algorithm (Pruesse et al., 2007) and the SILVA database version 132 (Quast et al., 2013). All sequences retrieved in this study were uploaded to European Nucleotide Archive (https://www.ebi.ac.uk/ena) submission number PRJEB32237.

### Class enrichment plot data preparation

**Figure 2:**
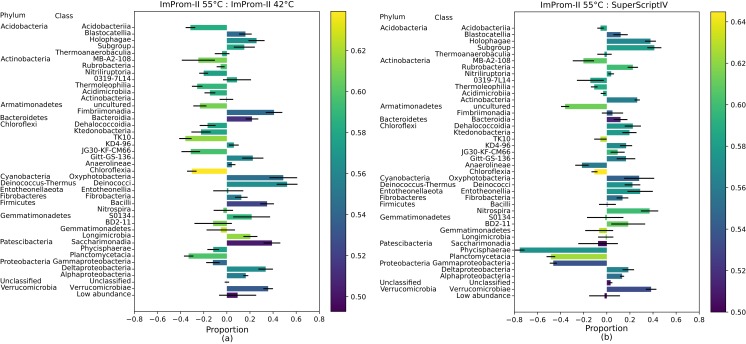
A proportional comparison of most abundant classes between the ImProm-II at 42 and 55 °C (A) and the ImProm-II at 55 °C and SuperScriptIV at 55 °C (B). Enrichment is expressed such that a class that is equally proportional in both conditions, has a value of 0. If the class shows in one condition but is absent from another, its value would be equal to 1 or −1 respectively. Furthermore, a weighted average of the GC content of each class is expressed as the bar color. Each value is an average of four biological replicates.

To explore whether G-C content contributed to differences in relative abundances of different taxa, we ran the following analysis: for each reaction condition we calculated pairwise comparisons at the class level: we normalized the proportional enrichment in each respective reaction conditions following Eq. (1) (Fig. 2; Fig. S2), where the A and B represent a class at the different conditions.

(1) $${\rm{Clas}}{{\rm{s}}_{{\rm{normalized}}}} = {{{\rm{A}} - {\rm{B}}} \over {{\rm{A}} + {\rm{B}}}}$$

The error bars represent a standard deviation, which have been calculated as a standard deviation of each category and normalized according to the Eq. (2). The δA and δB represent the standard deviation of class A and B. Details of deriving this equations are specified in Equation_S1 and Code_S3.ipynb.

(2) $${\rm{\delta Clas}}{{\rm{s}}_{{\rm{normalized}}}} = {2 \over {{{\left( {{\rm{A}} + {\rm{B}}} \right)}^2}}}\sqrt {{{\rm{B}}^2}{\rm{\delta }}{{\rm{A}}^2} + {{\rm{A}}^2}{\rm{\delta }}{{\rm{B}}^2}} $$

### Statistical analysis

All data analysis was performed in R v3.4.3 (R Core Team, 2018). The dataset was sub-sampled (rarified) to an even depth of 9,000 sequences per sample using python numpy package v1.15.4 (Van Der Walt, Colbert & Varoquaux, 2011). The subsampling removed five samples from the dataset. Additionally, three more samples were removed as outliers (Code_S1.ipynb). In order to equalize the number of replicates, two random samples were removed from the TGIRT dataset. This reduced the number of replicates to four samples per experimental category. The sample diversity was analyzed using the vegan package v2.5-2 (Oksanen et al., 2018). The data was visualized using the R package ggplot2 package v2.2.1 (Wickham, 2016) and python package matplotlib (Hunter, 2007).

## Results

### Sample preparation and diversity analysis

After removing obvious outliers, four samples from each condition were analyzed. The summary of the analysis and all the code used to produce each figure is included in the Files S1 and S2. The changes in bacterial diversity among the tested conditions were expressed using species count, Pielou’s evenness index (Pielou, 1966) and Shannon diversity index (Shannon & Weaver, 1949). Despite observed trends in the diversity indices, no statistically significant differences were detected (Fig. S1; Code_S2.ipynb).

### Relative abundance plot

A relative abundance of major taxonomic classes is depicted in the Fig. 1. Each column is an average of four biological replicates. OTUs that were not taxonomically assigned at this level are summarized as “Unclassified” (≈2%). Various patterns (detailled below) were detected among the experimental conditions: some patterns could be attributed to differences in reaction temperature (which ranged from 42 to 55 °C and 57 °C). Other patterns could be linked to enzyme type. RT reactions with SupeScript IV and TGIRT RT enzymes yielded no significant differences in class relative abundances. However, transcription with ImProm-II RT at a similar temperature (≈55 °C) yielded different abundances: specifically, the abundances of Alphaproteobacteria, Bacteroidia, Deltaproteobacteria, Oxyphotobacteria, Rubrobacteria, and Verrucomicrobidae decreased. However, Chloflexia, Gammaproteobacteria and Thermoleophilia abundances increased when their ribosomes were transcribed with ImProm-II RT (Fig. 1; Table S1). When transcription occured at lower temperature (42 °C), relative abundances of Bacilli, Deltaproteobacteria, and Oxyphotobacteria were enriched, while Actinobacteria, Chloroflexia, and Acidimicrobia were depleted under the same conditions (Fig. 1; Table S1).

### Class enrichment plot

The Fig. 2 also depicts the weighted average of the G-C content in each class. The proportional comparison is interpreted as follows: A value of zero in the proportional comparison represents the taxonomic class count that is exactly equal between the two compared groups (Fig. 2). A value of 1 or −1 is assigned when a given taxonomic class is only present in one category and absent in another, respectively. In general, there was a tendency toward lower G-C content lower temperature of ImProm-II (Fig. 2A; Figs. S2A and S2B). No statistically significant differences were detected between the profiles resulting from RT of SuprScript-IV or TGIRT (Fig. S2D). The taxa Gemmatimonadetes, Fibrobacteria, and Thermoanaerobaculia were relatively insensitive to the RT conditions. However the majority of the classes were enriched in some conditions. (I) the rate of RT reaction was only sensitive to temperature for the classes Alphaproteobacteria, Gemmatimonadetes, Fibrobacteria, Thermoanaerobaculia, TK10, and Blastocatellia (Fig. 2A). These groups tend to have extreme GC content (both high and low). (II) Gammaproteobacteria, Planctomycetacia, and Phycisphaerae are relatively insensitive to the reaction temperature (Fig. 2A), but their abundances vary with different RT enzymes (Fig. 2B; Fig. S2).

### Class enrichment statistics

We calculated a linear regression, where the response variable was the relative proportion of each class between two tested categories, and the explanatory variable was the G-C content (the assumptions tests and plots can be found in the File S2). The linear regression assumptions were tested: in case of two condition pairs (ImProm-II at 42 °C & SuperScript-IV as well as ImProm-II at 55 °C & SuperScript-IV), the assumption conditions were not met (File S2). Since we cannot confidently discard the null hypothesis in these cases, we do not consider these two tests significant. Therefore, we are considering only the ImProm-II 42 °C & ImProm-II 55 °C as well as ImProm-II 42 °C & TGIRT as a significant outcome. Differences in the remaining cases cannot be explained by the weighted G-C content alone (Table 3).

**Table 3:** The linear regression statistics.

Condition 1	Condition 2	Adjusted *R*^2^	*t* value	*p*-value	Significance	Note
ImProm-II 42 °C	ImProm-II 55 °C	0.429	5.366	4.89E-06	***	
ImProm-II 42 °C	SuperScript IV	0.1373	2.692	0.0127	*	•
ImProm-II 42 °C	TGIRT	0.2032	3.231	0.00264	**	
ImProm-II 55 °C	SuperScript IV	0.0841	−2.097	0.0431	*	•
ImProm-II 55 °C	TGIRT	0.03624	−1.546	0.131		
SuperScript IV	TGIRT	0.03634	1.548	0.130		

We used a GC content as an explanatory variable of a the class enrichment. The rows marked with a • do not fulfill all test assumptions (see Code_S2.ipynb). The statistical significance: *, **, *** at *P* < 0.05, 0.01 and 0.001 respectively.

## Discussion

As high throughput sequencing has become increasingly accessible in recent years, researchers urgently call for method standardization to allow for accurate cross-study comparisons (Pan et al., 2010; Blagodatskaya & Kuzyakov, 2013). With this motivation, researchers developed new platforms that offer protocols and standardized methods for data acquisition from DNA resources, such as the Earth Microbiome Project (http://www.earthmicrobiome.org/) that standardizes DNA amplicon sequencing. Despite the success with standardizing methods and protocols for DNA-based analysis, to this date, there is RNA-based methods have not been standardized, despite the discrepancies repeatedly reported between the RNA- and DNA-based analyses (Blazewicz et al., 2013; Carini et al., 2016; Dlott et al., 2015) and the plethora of methods used in these studies (Table 1). The analysis of rRNA adds specific biases to high throughput sequencing analysis, such as reduced template stability compared to DNA, RT priming bias, and linearity of RT reaction (Bustin & Nolan, 2004). These biases need to be either minimized or standardized.

In this study, we focused on one crucial step in the RNA analysis that was previously overlooked: the transcription of RNA to cDNA (Table 1). Several biases connected to the RT reaction have been described for RT-qPCR, such as quantification of expressed genes (Bustin & Nolan, 2004, 2017; Zhang & Byrne, 1999) in RNA-Seq, that is, primer related bias of expressed transcripts (Hansen, Brenner & Dudoit, 2010). Yet, the role of RT in diversity patterns was not yet investigated in the context of high throughput sequencing of ribosomes. Here, we focus on the role of enzyme and reaction temperature in shaping the diversity and composition of ribosome-based communities. We have compared four RT enzymes commonly used in soil microbiology (Table 1) at two distinct temperature modes (42 °C and 55–57 °C). Then we analyzed temperature and RT enzyme-related effects on the resulting community profiles (Table 2).

Under different reaction conditions, we detected differences in the relative abundance of bacterial classes portrayed by different reaction conditions (Fig. 1). Some observed differences can be attributed to the combined effect of reaction temperature and average template G-C content (Fig. 2; Fig. S2). As expected, this effect is clearest when we applied the same enzyme (ImProm-II) at two reaction temperatures (42 and 55 °C), then the G-C content had the highest prediction power (*t* = 5.366, *p* = 4.8 × 10^−5^, Table 3). Likewise, in every comparison of RT enzymes at low and high temperature, G-C content affected the relative abundance of taxonomic classes with statistical significance. Although the RT reactions are commonly performed at 42 °C (Table 1), our results indicate that this reaction temperature is too low to allow successful RT of some soil community taxa, in particular species with higher G-C content.

When transcription was performed with different enzymes under similar reaction temperatures, relative abundances of taxa differed notably (Figs. 1 and 2). Although the RTs of SuperScript-IV and TGIRT originate from different organisms, they yielded similar taxa abundances. Reactions with ImProm-II yielded different profiles. These differences cannot be explained by the G-C content (Table 3), but could be attributed to ribosome properties and the efficiency of RT. The ribosomes extracted from the soil environment were diverse and probably differ in their secondary and tertiary structures (Yilmaz, Okten & Noguera, 2006) and post-transcriptional modifications (Schwartz & Motorin, 2017). Thus RT enzymes kinetics would differ. The discrepancies reported in this study raise further questions: how would one decide which enzymes or temperatures best reflect the active community composition? It has been suggested that the total community could be used as a reference to accurately deduce the diversity. Furthermore, this study was performed on desert soil samples and the effects in other environments remain to be determined.

We previously demonstrated that the total and active communities in the Negev soil used in this study differ in both abundance profiles and community composition during the dry season, while during the wet season, no differences were detected (Baubin et al., 2019). During the dry season, a “phantom taxa” (Klein et al., 2016), Deinococcus-Thermus, comprised ≈30% of the total soil community (Baubin et al., 2019) but was undetected in the active community of the dry season by any of the methods used here. These results suggest that the DNA-based total community may differ from the RNA-based community and thus cannot be used as a reliable reference for diversity. Furthermore, our results underline a need to standardize and specify the RT conditions that allow cross-study comparisons. The scale of the effect of RT conditions on the RNA-based community might vary with a studied biome. Dependending on obvious factors such as GC content (discussed above), as well as poorly studied factors such as ribosomal post-transcriptional modifications. Therefore, we recommend verifying each case separately before attempting a cross-study comparison.

## Conclusion

We have tested commonly utilized RT enzymes at assorted temperatures and observed marked differences in the output community structure. These differences were attributed to RT type and reaction conditions. We suggest that RT reaction conditions may dictate the diversity of a given community and therefore the exact conditions should be detailed in full (i.e., the common notation, “according to manufacturer instructions” does not provide sufficient information (Table 1)). Furthermore, RT should be performed at a sufficiently high temperature to minimize the G-C bias, preferably at 55 °C. Lastly, we suggest that the same RT enzyme should be used across comparable studies, since we detected discrepancies between RT enzymes performing at equivalent conditions (Fig. 2B). Here, we highlight, for the first time, the need for standardisation and careful consideration of RT reaction conditions in studies describing ribosome-based diversity and community composition.

## Supplemental Information

10.7717/peerj.7608/supp-1 Supplemental Information 1 Diversity, richness and evenness across experimental conditions. Diversity plot displaying species richness, Pelou evenness, and Shannon diversity for a variety of experimental conditions (X-axis). Species richness is represented as species count. Each category is an average of 4 biological replicates.

10.7717/peerj.7608/supp-2 Supplemental Information 2 A proportional relation of most abundant classes and their respective G-C contents between the tested conditions. A comparison between the ImProm-II at 42 ° C and SuperScriptIV (a),the ImProm-II at 55 ° C and TGIRT, (b), ImProm-II at 42 ° C and TIGRT (c) and TGIRT and SuperScriptIV (d). Enrichment is expressed such that a class that is equally proportional in both conditions, has a value of 0. If the class shows in one condition, but its absent from another, its value would be equal to 1 or -1 respectively. Furthermore a weighted average of the GC content of each class is expressed as the bar color. Each value is an average of 4 biological replicates.

10.7717/peerj.7608/supp-3 Supplemental Information 3 Relative abundance of phyla across the experimental conditions. Each column is an average of 4 biological replicates.

10.7717/peerj.7608/supp-4 Supplemental Information 4 Deriving formula for error propagation through the normalization formula used in Figure 2 and Figure S2. The formula compares taxonomic classes relative enrichment between two experimental conditions as well as scaling. The classes are expressed as A and B and the standard error in the formula corresponds to.

10.7717/peerj.7608/supp-5 Supplemental Information 5 Data preparation IPython notebook code. Details of the sequencing data preparation procedure, including the discarding of outlier samples and normalization of sequence counts.

10.7717/peerj.7608/supp-6 Supplemental Information 6 Diversity statistics code file iPython notebook. A detailed description of the procedure of generating the diversity statistical comparison between the tested conditions.

10.7717/peerj.7608/supp-7 Supplemental Information 7 GC enrichment plot IPython notebook code. A detailed description of the code used to generate the GC enrichment figure.

## Additional Information and Declarations

### DNA Deposition

The following information was supplied regarding the deposition of DNA sequences: The sequences retrieved in this study are available at the European Nucleotide Archive: PRJEB32237.

### Data Availability

The following information was supplied regarding data availability: The code used to generate the data is available in the Supplemental Files.
