# Supplementary material for: The effect of reverse transcription enzymes and conditions on high throughput amplicon sequencing of the 16S rRNA
Source: PeerJ. 2019 Oct 25;7:e7608. doi: 10.7717/peerj.7608 (PMC6816399; doi:10.7717/peerj.7608)
Supplement: Supplemental Information 1 — Diversity plot displaying species richness, Pelou evenness, and Shannon diversity for a variety of experimental conditions (X-axis). Species richness is represented as species count. Each category is an average of 4 biological replicates. [file peerj-07-7608-s001.pdf]

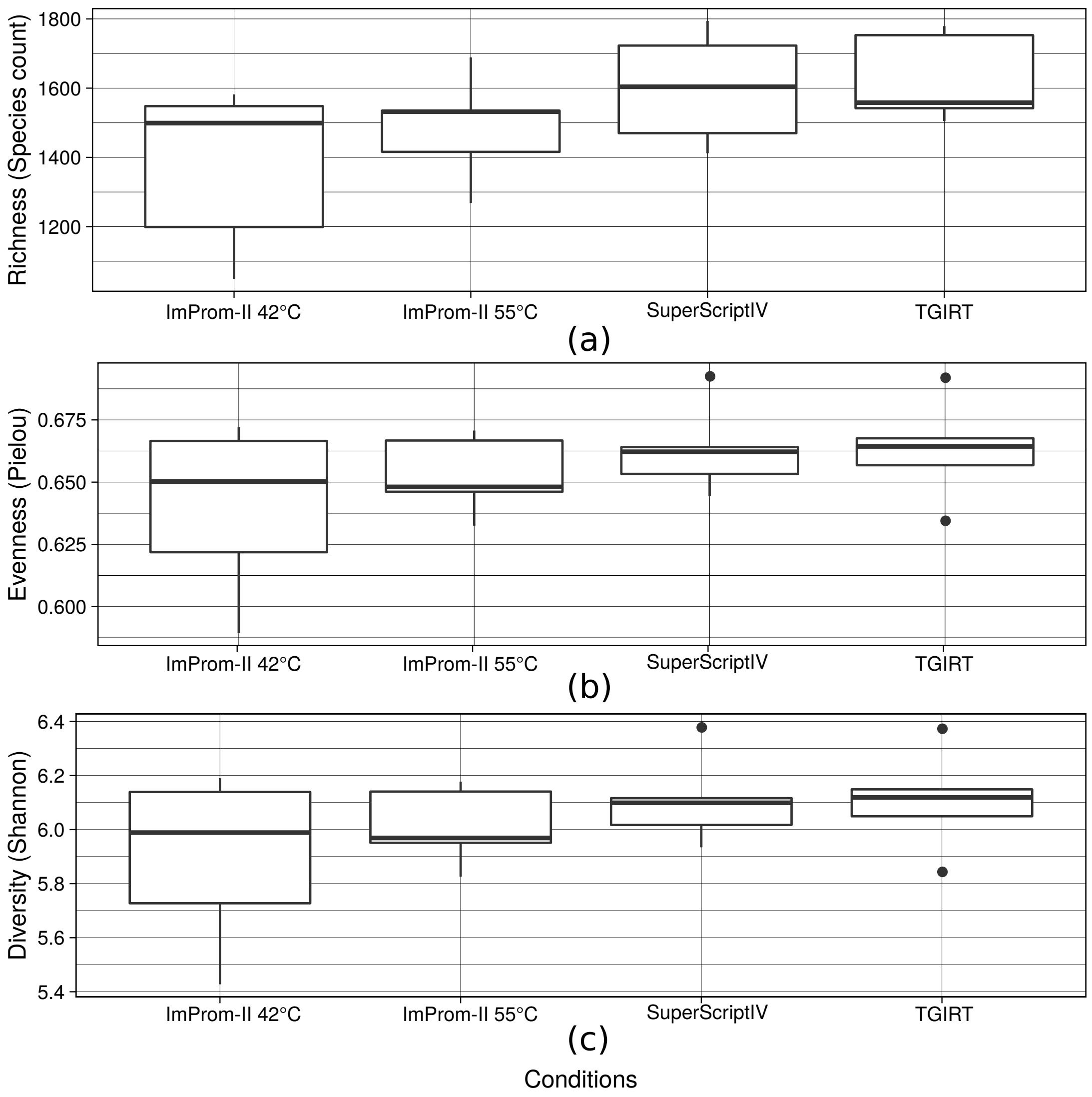


**Figure S1.** Diversity plot displaying species richness, Pelou evenness, and Shannon diversity for a variety of experimental conditions (X-axis). Species richness is represented as species count. Each category is an average of 4 biological replicates.
