# Supplementary material for: The effect of reverse transcription enzymes and conditions on high throughput amplicon sequencing of the 16S rRNA
Source: PeerJ. 2019 Oct 25;7:e7608. doi: 10.7717/peerj.7608 (PMC6816399; doi:10.7717/peerj.7608)
Supplement: Supplemental Information 2 — A comparison between the ImProm-II at 42 ° C and SuperScriptIV (a),the ImProm-II at 55 ° C and TGIRT, (b), ImProm-II at 42 ° C and TIGRT (c) and TGIRT and SuperScriptIV (d). Enrichment is expressed such that a class that is equally proportional in both conditions, has a value of 0. If the class shows in one condition, but its absent from another, its value would be equal to 1 or -1 respectively. Furthermore a weighted average of the GC content of each class is expressed as the bar color. Each value is an average of 4 biological replicates. [file peerj-07-7608-s002.pdf]

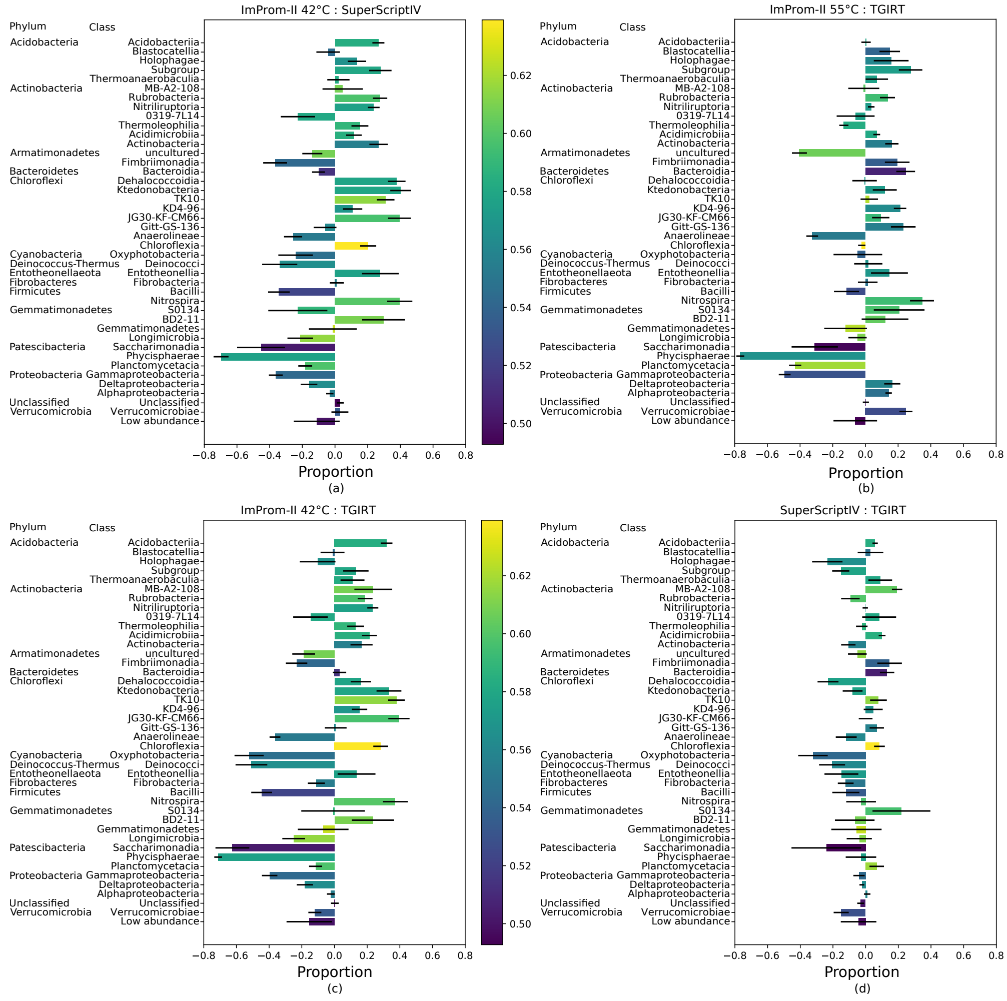

Figure S2. A proportional comparison of most abundant classes between the ImProm-II at 42 °C and SuperScriptIV (a),the ImProm-II at 55 °C and TGIRT, (b), ImProm-II at 42 °C and TIGRT (c) and TGIRT and SuperScriptIV (d). Enrichment is expressed such that a class that is equally proportional in both conditions, has a value of 0. If the class shows in one condition, but its absent from another, its value would be equal to 1 or -1 respectively. Furthermore a weighted average of the GC content of each class is expressed as the bar color. Each value is an average of 4 biological replicates with the error bars representing the scaled standard deviation.
