# Supplementary material for: The effect of reverse transcription enzymes and conditions on high throughput amplicon sequencing of the 16S rRNA
Source: PeerJ. 2019 Oct 25;7:e7608. doi: 10.7717/peerj.7608 (PMC6816399; doi:10.7717/peerj.7608)
Supplement: Supplemental Information 3 — Each column is an average of 4 biological replicates. [file peerj-07-7608-s003.docx]

Supplementary table 1: Relative abundance of phyla across the experimental conditions. Each column is an average of 4 biological replicates.

| Enzyme | ImProm-II 42°C | | ImProm-II 55°C | SuperScriptIV | TGIRT |
| --- | --- | --- | --- | --- | --- |
| Class |  | |  |  |  |
| 0319-7L14 | 527 | | 442 | 332 | 392 |
| ABY1 | 0 | 0 | | 0 | 0 |
| AKAU4049 | 0 | | 1 | 3 | 4 |
| Acidimicrobiia | 1203 | | 1617 | 1518 | 1858 |
| Acidobacteriia | 257 | | 259 | 444 | 359 |
| Actinobacteria | 5864 | | 11263 | 10105 | 11353 |
| Alphaproteobacteria | 12943 | | 9302 | 12148 | 12385 |
| Anaerolineae | 191 | | 175 | 113 | 89 |
| Armatimonadia | 6 | | 19 | 1 | 2 |
| BD2-11 | 26 | | 33 | 48 | 42 |
| BD7-11 | 7 | | 4 | 4 | 0 |
| Babeliae | 11 | | 10 | 18 | 9 |
| Bacilli | 1356 | | 656 | 663 | 520 |
| Bacteroidia | 2825 | | 1817 | 2307 | 3005 |
| Berkelbacteria | 2 | | 0 | 1 | 0 |
| Blastocatellia | 336 | | 243 | 309 | 328 |
| Chlamydiae | 25 | | 23 | 6 | 3 |
| Chloroflexia | 441 | | 825 | 666 | 789 |
| Chthonomonadetes | 0 | | 0 | 0 | 0 |
| Clostridia | 13 | | 13 | 8 | 16 |
| Coriobacteriia | 1 | | 0 | 0 | 0 |
| Dehalococcoidia | 70 | | 98 | 155 | 97 |
| Deinococci | 89 | | 28 | 44 | 29 |
| Deltaproteobacteria | 4330 | | 2154 | 3141 | 3001 |
| Elusimicrobia | 6 | | 0 | 2 | 2 |
| Entotheonellia | 125 | | 122 | 221 | 164 |
| Erysipelotrichia | 0 | | 1 | 5 | 0 |
| FFCH5909 | 0 | | 0 | 1 | 0 |
| Fibrobacteria | 45 | | 35 | 46 | 36 |
| Fimbriimonadia | 69 | | 29 | 32 | 43 |
| Fusobacteriia | 1 | | 0 | 0 | 0 |
| Gammaproteobacteria | 5967 | | 7610 | 2793 | 2576 |
| Gemmatimonadetes | 532 | | 591 | 518 | 463 |
| Gitt-GS-136 | 270 | | 171 | 239 | 274 |
| Holophagae | 235 | | 139 | 308 | 191 |
| Hydrogenedentia | 0 | | 2 | 1 | 0 |
| Ignavibacteria | 31 | | 22 | 37 | 32 |
| JG30-KF-CM66 | 76 | | 145 | 175 | 175 |
| KD4-96 | 298 | | 264 | 370 | 406 |
| Ktedonobacteria | 26 | | 41 | 61 | 52 |
| Lineage | 27 | | 9 | 5 | 8 |
| Longimicrobia | 328 | | 217 | 213 | 197 |
| MB-A2-108 | 61 | | 101 | 67 | 99 |
| Melainabacteria | 15 | | 3 | 13 | 9 |
| Methanomicrobia | 2 | | 0 | 0 | 0 |
| Microgenomatia | 9 | | 4 | 0 | 5 |
| Mollicutes | 0 | | 4 | 0 | 0 |
| NC10 | 3 | | 4 | 7 | 10 |
| Negativicutes | 10 | | 4 | 0 | 0 |
| Nitriliruptoria | 253 | | 380 | 410 | 408 |
| Nitrososphaeria | 0 | | 12 | 1 | 0 |
| Nitrospira | 32 | | 34 | 74 | 70 |
| OLB14 | 23 | | 24 | 44 | 56 |
| OM190 | 0 | | 0 | 0 | 0 |
| Oxyphotobacteria | 2798 | | 962 | 1711 | 877 |
| P2-11E | 7 | | 3 | 3 | 3 |
| Parcubacteria | 3 | | 1 | 0 | 2 |
| Phycisphaerae | 101 | | 128 | 18 | 17 |
| Pla4 | 8 | | 4 | 3 | 8 |
| Planctomycetacia | 357 | | 710 | 247 | 283 |
| Rhodothermia | 0 | | 11 | 10 | 5 |
| Rubrobacteria | 1351 | | 1505 | 2380 | 1974 |
| S0134 | 116 | | 75 | 73 | 114 |
| SHA-26 | 4 | | 12 | 16 | 4 |
| Saccharimonadia | 130 | | 57 | 49 | 30 |
| Sericytochromatia | 53 | | 25 | 33 | 27 |
| Spirochaetia | 0 | | 1 | 0 | 0 |
| Subgroup | 385 | | 284 | 681 | 501 |
| Synergistia | 0 | | 0 | 0 | 0 |
| TK10 | 275 | | 583 | 522 | 611 |
| Thermoanaerobaculia | 117 | | 127 | 122 | 146 |
| Thermodesulfovibrionia | 0 | | 0 | 0 | 0 |
| Thermoleophilia | 2745 | | 4643 | 3733 | 3562 |
| Thermoplasmata | 14 | | 14 | 5 | 4 |
| Unclassified | 1211 | | 1210 | 1292 | 1216 |
| Verrucomicrobiae | 1310 | | 620 | 1391 | 1029 |
| WS6 | 0 | | 0 | 2 | 1 |
| WWE3 | 0 | | 0 | 0 | 0 |
| Woesearchaeia | 0 | | 2 | 0 | 0 |
| uncultured | 41 | | 66 | 31 | 28 |
| vadinHA49 | 7 | | 7 | 1 | 1 |
