# Supplementary material for: The effect of reverse transcription enzymes and conditions on high throughput amplicon sequencing of the 16S rRNA
Source: PeerJ. 2019 Oct 25;7:e7608. doi: 10.7717/peerj.7608 (PMC6816399; doi:10.7717/peerj.7608)
Supplement: Supplemental Information 4 — The formula compares taxonomic classes relative enrichment between two experimental conditions as well as scaling. The classes are expressed as A and B and the standard error in the formula corresponds to. [file peerj-07-7608-s004.pdf]

$$f = \frac{A-B}{A+B},$$

$$f = \frac{A}{(A+B)} - \frac{B}{A+B}$$

$$\delta f = \sqrt{\left(\frac{\partial f}{\partial A}\delta A\right)^2 + \left(\frac{\partial f}{\partial B}\delta B\right)^2}$$

$$\frac{\partial f}{\partial A} = \frac{1(A+B)-A}{(A+B)^2} - \frac{-B}{(A+B)^2} = \frac{2B}{(A+B)^2}$$

$$\frac{\partial f}{\partial B} = \frac{-A}{(A+B)^2} - \frac{1(A+B)B}{(A+B)^2} = \frac{-2A}{(A+B)^2}$$

$$\delta f = \sqrt{\left(\frac{2B}{(A+B)^2}\delta A\right)^2 + \left(-\frac{2A}{(A+B)^2}\delta B\right)^2}$$

$$\delta f = \frac{2}{(A+B)^2}\sqrt{B^2\delta A^2 + A^2\delta B^2}$$

Equation S1. Finding the formula for error propagation through the normalization formula used to calculate pairwise comparisons depicted in Figure 2 and Figure S2. The formula compares taxonomic classes relative enrichment between two experimental conditions as well as scaling. The classes are expressed as A and B and the standard error corresponds to δA and δB respectively. The resulting normalized standard deviation is represented as δf.
